# Supplementary material for: A Critical View of the Application of the APEX Software (Aqueous Photochemistry of Environmentally-Occurring Xenobiotics) to Predict Photoreaction Kinetics in Surface Freshwaters
Source: Molecules. 2019 Dec 18;25(1):9. doi: 10.3390/molecules25010009 (PMC7017383; doi:10.3390/molecules25010009)
Supplement: Supplementary file 1 [file molecules-25-00009-s001.zip › 3.2.4_gcc-4.4.0.zip!/3.2.4_gcc-4.4.0/3.2.4_gcc-4.4.0/doc/octave/html/interpreter/Trouble.html]

Trouble - Untitled


Next: Installation,
Previous: Contributing Guidelines,
Up: Top


---

## Appendix E Known Causes of Trouble

This section describes known problems that affect users of Octave. Most
of these are not Octave bugs per se—if they were, we would fix them.
But the result for a user may be like the result of a bug.

Some of these problems are due to bugs in other software, some are
missing features that are too much work to add, and some are places
where people's opinions differ as to what is best.

- Actual Bugs: Bugs we will fix later.- Reporting Bugs- Bug Criteria- Bug Lists- Bug Reporting- Sending Patches- Service
